# Supplementary material for: The interactome of intact mitochondria by cross-linking mass spectrometry provides evidence for coexisting respiratory supercomplexes
Source: Mol Cell Proteomics. 2017 Dec 8;17(2):216–32. doi: 10.1074/mcp.RA117.000470 (PMC5795388; doi:10.1074/mcp.RA117.000470)
Supplement: Supplemental Data [file supp_RA117.000470_133922_0_supp_23978_fzffwf.zip › spectra_annotation/mito_DR_spectra_annotation/7-1-4-1-10-1.pdf]

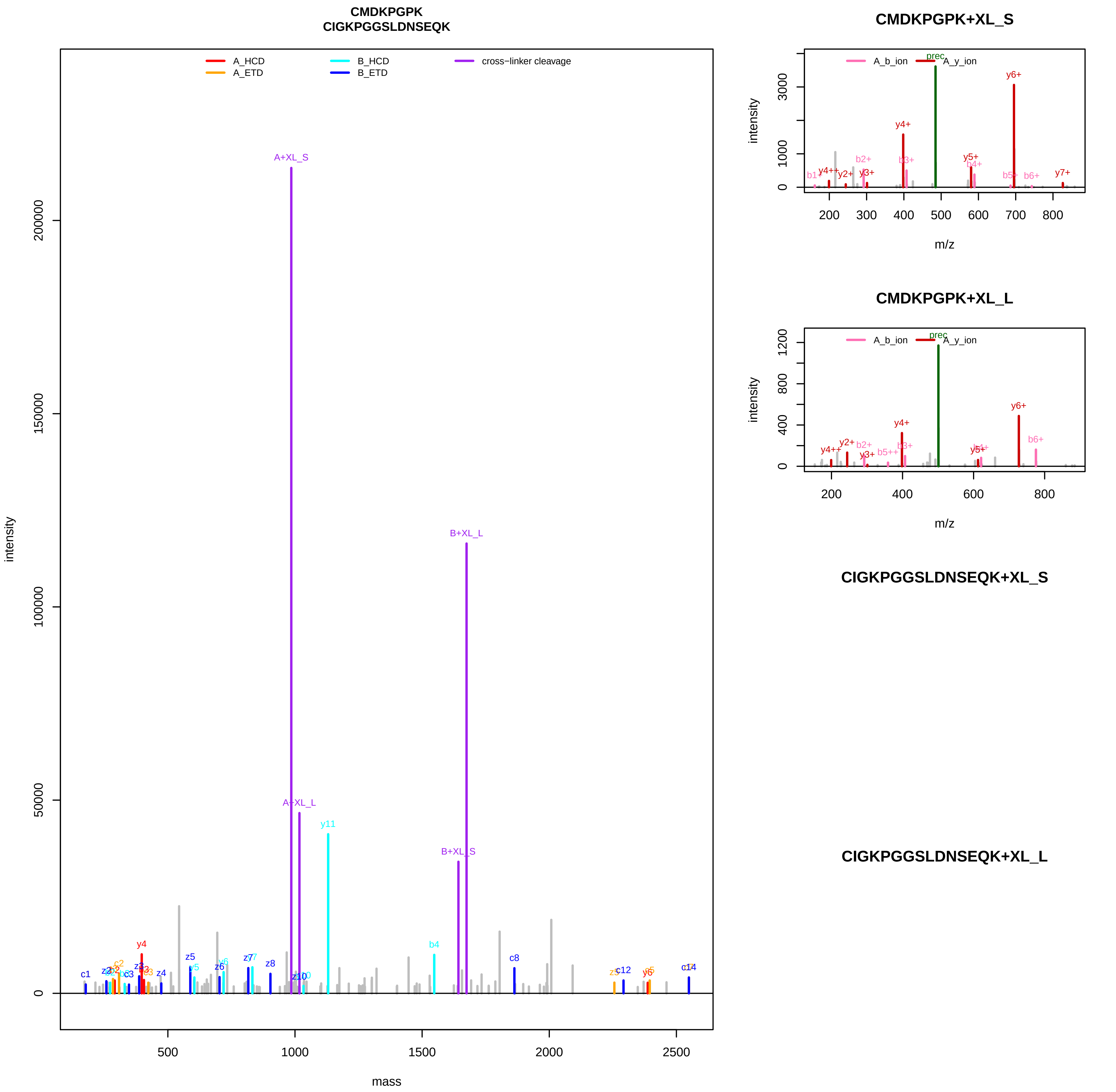
CMDKPGPK
CIGKPGGSLDNSEQK
A_HCD
A_ETD
B_HCD
B_ETD
cross−linker cleavage
A+XL_S
A+XL_L
B+XL_L
B+XL_S
y11
y4
b4
c8
z8
z5
z6
z7
c12
c14
intensity
mass
0
50000
100000
150000
200000
500
1000
1500
2000
2500
CMDKPGPK+XL_S
A_b_ion
A_y_ion
prec
y6+
y4+
y5+
y7+
b2+
b3+
b4+
b5+
b6+
y2+
y3+
b1+
y4++
intensity
m/z
CMDKPGPK+XL_L
A_b_ion
A_y_ion
prec
y6+
y4+
b6+
y2+
b2+
b3+
y3+
b5++
y4++
y5+
intensity
m/z
CIGKPGGSLDNSEQK+XL_S
CIGKPGGSLDNSEQK+XL_L
